# Development and Piloting of Co.Ge.: A Web-Based Digital Platform for Generative and Clinical Cognitive Assessment

**DOI:** 10.3390/jpm15090423

**Published:** 2025-09-03

**Authors:** Angela Muscettola, Martino Belvederi Murri, Michele Specchia, Giovanni Antonio De Bellis, Chiara Montemitro, Federica Sancassiani, Alessandra Perra, Barbara Zaccagnino, Anna Francesca Olivetti, Guido Sciavicco, Rosangela Caruso, Luigi Grassi, Maria Giulia Nanni

**Affiliations:** 1 Institute of Psychiatry, Department of Neuroscience and Rehabilitation, University of Ferrara, 44121 Ferrara, Italy; angela.muscettola@unife.it (A.M.); giovanniantonio.debellis@unife.it (G.A.D.B.); chiara.montemitro@unife.it (C.M.); barbara.zaccagnino@edu.unife.it (B.Z.); annafrancesca.olivetti@gmail.com (A.F.O.); rosangela.caruso@unife.it (R.C.); luigi.grassi@unife.it (L.G.); mariagiulia.nanni@unife.it (M.G.N.); 2 Integrated Department of Mental Health and Pathological Addictions, Local Health Trust of Ferrara, 44124 Ferrara, Italy; michele.specchia@edu.unife.it; 3 Department of Medical Sciences and Public Health, University of Cagliari IT, 09124 Cagliari, Italy; federicasancassiani@yahoo.it (F.S.); alessandra.perra@unica.it (A.P.); 4 Department of Mathematics and Computer Science, University of Ferrara, 44121 Ferrara, Italy; guido.sciavicco@unife.it

**Keywords:** computational psychiatry, digital psychiatry, cognitive, neuropsychology, reaction time, Bayesian, verbal memory, precision medicine, tailored cognitive batteries, generative modeling

## Abstract

**Background/Objectives**: This study presents Co.Ge. a Cognitive Generative digital platform for cognitive testing. We describe its architecture and report a pilot study. **Methods**: Co.Ge. is modular and web-based (Laravel-PHP, MySQL). It can be used to administer a variety of validated cognitive tests, facilitating administration and scoring while capturing Reaction Times (RTs), trial-level responses, audio, and other data. Co.Ge. includes a study-management dashboard, Application Programming Interfaces (APIs) for external integration, encryption, and customizable options. In this demonstrative pilot study, clinical and non-clinical participants completed an Auditory Verbal Learning Test (AVLT), which we analyzed using accuracy, number of recalled words, and reaction times as outcomes. We collected ratings of user experience with a standardized rating scale. Analyses included Frequentist and Bayesian Generalized Linear Mixed Models (GLMMs). **Results**: Mean ratings of user experience were all above 4/5, indicating high acceptability (*n* = 30). Pilot data from AVLT (*n* = 123, 60% clinical, 40% healthy) showed that Co.Ge. seamlessly provides standardized clinical ratings, accuracy, and RTs. Analyzing RTs with Bayesian GLMMs and Gamma distribution provided the best fit to data (Leave-One-Out Cross-Validation) and allowed to detect additional associations (e.g., education) otherwise unrecognized using simpler analyses. **Conclusions**: The prototype of Co.Ge. is technically robust and clinically precise, enabling the extraction of high-resolution behavioral data. Co.Ge. provides traditional clinical-oriented cognitive outcomes but also promotes complex generative models to explore individualized mechanisms of cognition. Thus, it will promote personalized profiling and digital phenotyping for precision psychiatry and rehabilitation.

## 1. Introduction

Cognitive performance affects individual autonomy, treatment adherence, and functional outcomes across clinical and non-clinical populations. Cognitive assessments are central to the evaluation of numerous conditions, including neurodegenerative diseases, psychiatric disorders, and acquired brain injuries. Standardized neuropsychological tests play a key role in identifying impairments, tracking progression, and informing clinical decisions [1,2,3].

Traditionally, cognitive assessments are conducted using paper-based protocols administered by trained professionals. Widely used screening tools such as the Mini-Mental State Examination (MMSE) and the Montreal Cognitive Assessment (MoCA), as well as comprehensive neuropsychological batteries, are well validated and broadly adopted. While such tools are well validated and widely adopted, they are labor-intensive, susceptible to scoring variability, and limited in their ability to capture detailed behavioral information such as response times, error types, and trial-level variability [4]. In addition, analogue data collection constrains structured storage, scalability, and integration with digital health systems. Recent systematic reviews confirm that digital cognitive instruments can achieve diagnostic performance comparable to or better than traditional tests, with several tools reporting sensitivities and specificities above 0.80 for mild cognitive impairment and dementia. However, existing platforms remain heterogeneous: Some are digital translations of traditional tasks (e.g., computerized MoCA, clock drawing), others are revised versions (e.g., CNS Vital Signs, CogState, BrainCheck, MemTrax), and others represent novel paradigms (e.g., SATURN, Brain Health Assessment). Despite these advances, only a subset of available computerized cognitive instruments—like CNS Vital Signs [5], CogState [6], BrainCheck [7], and MemTrax [8]—meet acceptable standards for reliability, sensitivity, and ecological validity in applied clinical settings. Others continue to require costly devices or professional supervision, or lack robust normative cut-offs and validation in diverse populations, which limits their broader clinical adoption [9,10].

In recent years, there has been growing demand for scalable, standardized, and data-rich assessment tools. This has led to the development of digital cognitive platforms that combine psychometric rigor with enhanced accessibility and automation [11]. Computerized testing offers several advantages, including standardized administration, automatic scoring, and the ability to collect high-resolution behavioral metrics. Studies have also demonstrated the feasibility and acceptability of online cognitive testing, including among older adults and clinical populations [12,13].

Despite these advances, many digital platforms remain limited in scope, focusing narrowly on specific domains or lacking interoperability with clinical infrastructures and research pipelines [4,10]. In particular, it would be desirable to integrate digital cognitive tests with computational and/or generative cognitive models because of their potential to infer latent mechanisms and develop personalized assessments and interventions, although their psychometric properties are not yet firmly established [14,15,16]. Thus, in order to maximize effectiveness both in clinical and in translational contexts, digital tools should yield detailed behavioral data suitable for advanced modeling while retaining some level of compatibility with conventional scoring formats that are familiar to clinicians. This “dual use” would be essential to bridge traditional assessment with computational approaches and ensure comparability with the large body of literature grounded in standardized cognitive testing.

Here, we present an overview of the Cognitive Generative (Co.Ge.) platform, describing its architecture and functionalities. We also showcase preliminary data from its initial clinical deployment, from an Auditory Verbal Learning Test (AVLT). We discuss potential applications of Co.Ge. in routine cognitive assessment as well as in computational modeling, with a focus on its capacity to generate structured, high-resolution behavioral datasets for personalized cognitive assessments.

## 2. Materials and Methods

### 2.1. Test Development and Digital Platform

#### 2.1.1. Platform Design and Objectives

The Co.Ge. platform is a modular, web-based tool developed within the broader DK (Data and Knowledge) project, which aims to improve the collection, integration, and digital management of biomedical data. DK is an institutional initiative supporting the development of secure, interoperable infrastructures for biomedical research and clinical workflows. Co.Ge. is specifically designed to digitalize the administration, scoring, and data handling of various neuropsychological tests. It can accommodate the structure, content, and scoring logic of validated paper-based protocols while enhancing usability through automated administration, secure data storage, and structured output.

At the moment, the platform supports examiner-supervised testing through an interactive browser interface, preserving clinical oversight while enabling automated scoring and real-time data capture. Each task reproduces the structure and original instructions of its paper equivalent. Age- and education-based corrections are applied where relevant to ensure interpretability according to normative references [17,18,19].

Besides computing standard scoring, Co.Ge. records trial-level reaction times, error patterns, and—when applicable—verbal responses via audio. Data are stored in structured, exportable formats for input in statistical software and electronic health records. The system was built for clinical deployment and to support future application of generative cognitive models, enabling in-depth analyses of latent processes such as processing speed, attentional control, and decision dynamics [4,15].

The study was conducted in accordance with the Declaration of Helsinki and approved by Comitato Etico di Area Vasta Emilia Centro (protocol code 199/2023/Disp/AUSLFe, approved on 4 April 2023, data collection date: October 2024 and June 2025).

#### 2.1.2. Software Architecture and Technologies

Co.Ge. was developed using the Laravel PHP framework (v11.x), which provides an MVC (Model–View–Controller) architecture for scalable and modular design. Backend development relies on Laravel’s ecosystem, including packages such as Laravel/UI (authentication), the Maat website/Excel (data import/export), and spatie/Laravel-backup (automated backups). The frontend combines Blade templates, Bootstrap, and Core UI, with JavaScript components compiled via Vite.

Data are managed through 5.0 Laravel’s Eloquent ORM and stored in a MySQL (version 8.0.4) relational database, enabling structured representation of entities such as studies, users, and participants. This schema supports longitudinal designs and integration with other data collection platforms such as REDCap [20]. Authentication is managed via session-based Laravel UI scaffolding with role-based access control and CSRF protection. Passwords are encrypted using Bcrypt (6.0.0), and access is restricted to authorized users only [21].

#### 2.1.3. Dashboard and Study Management

The web-based dashboard allows authorized personnel to configure studies, enroll participants, assign test batteries, initiate sessions, and visualize outcomes. Test sessions are conducted on secure devices with examiner supervision and can follow either cross-sectional or longitudinal protocols.

Study metadata, participant information, and response data are linked via a relational structure that enables monitoring of progress and completeness. Test configurations allow for fixed or alternate test versions, repeated timepoints, and customized scheduling.

#### 2.1.4. Data Structure and Interoperability

The database schema was designed using Laravel migrations and comprises dedicated tables for each cognitive task. All sessions are time-stamped and indexed by anonymized alphanumeric participant codes. The model supports linkage with REDCap-based datasets and can export results in PDF, CSV, SQL, or JSON formats. An API is available for secure programmatic access.

All data are encrypted at rest and during transfer. Automated backups and validation routines (range checks, completeness, identifier uniqueness) are applied to ensure data integrity. Access to identifiable data is restricted in compliance with the GDPR and local clinical governance protocols.

### 2.2. Cognitive Battery

In its present version (1.0), Co.Ge. includes digital versions of widely used neuropsychological tests, each adapted to preserve the structure and scoring system of widely available formats. The current implementation includes the following tasks:

- *Digit Span (Forward/Backward)—DS/DSB*: Sequence repetition tasks with automated scoring and voice onset latency detection.
- *Trail Making Test (A/B)—TMT*: Timed sequencing tasks with real-time error tracking and completion time computation.
- *Stroop Test*: Three-phase task with automated comparison of verbal responses and expected outputs.
- *Rey Auditory Verbal Learning Test—AVLT*: Multi-trial word recall and recognition task with automated scoring and audio capture.
- *Phonemic and Semantic Verbal Fluency*: Timed free recall tasks recorded and analyzed for accuracy, repetitions, and rule violations.
- *Similarities*: Conceptual abstraction task scored for semantic relatedness.
- *Contrasting Instructions/Go–No-Go:* Inhibitory control tasks recording error types and reaction times.
- *Matrix and Estimation Tasks*: Timed visual and numerical estimation tests corrected for demographic variables.
- *Probabilistic Reward Task—PRT*: Reinforcement learning paradigm capturing accuracy, bias, and decision consistency across trials.

All tasks record trial-level events, reaction times, and, when relevant, audio or click-stream data. Scoring follows validated correction procedures [11].

### 2.3. Data Capture, Processing, and Export

All data collected through Co.Ge. are stored in a structured relational database. Data are stored securely on password-protected institutional servers in compliance with Italian law (GDPR).

Each cognitive task is linked to a specific MySQL table, managed through Laravel migrations, and relationally connected to core entities (Study, Patient, User) to enable cross-sectional and longitudinal tracking of individual performance.

During each session, the system captures (i) session metadata (e.g., participant code, examiner, timestamp); (ii) standard performance indices (e.g., raw scores, adjusted equivalents, completion time, error types); (iii) trial-level metrics, including reaction times, stimulus–response pairs, and latencies; and (iv) audio recordings for applicable tasks (e.g., Stroop, fluency, Rey), aligned with stimulus timing. A speech-to-text (STT) module is currently under development, which will rely on timestamp alignment and will be validated against expert-coded human transcriptions.

A dedicated audio module records verbal responses through the device’s microphone and stores each audio file securely, linked to the corresponding stimulus via timestamp alignment. At present, transcription is performed manually by trained raters to ensure precision and consistency, especially in clinical settings. A speech-to-text pipeline is under development to enable automated transcription and response labeling, which will later be validated against expert-coded transcripts to assess accuracy, robustness, and usability across populations with different cognitive and linguistic profiles.

Data are primarily collected automatically during task execution but can be manually completed via the dashboard in case of missing entries. Super-users can amend patient profiles, examiner assignments, or test logs via validated interface forms. Bulk import of participant ID’s is supported via Excel templates or REDCap integration. Export options include CSV, SQL, PDF, and structured formats for external synchronization. An API is available for secure, programmatic data access.

Automated validation routines are designed to flag missing fields or inconsistencies, while additional preprocessing (e.g., latency smoothing, verbal response annotation) can be performed via internal pipelines. High-resolution temporal traces (e.g., mouse clicks, screen transitions, voice onset) enable enriched data collection for future applications within generative models, such as drift diffusion, accumulator, or reinforcement learning frameworks.

The platform enforces data protection through encryption, automated backups, and role-based access controls. Only authorized personnel can access or modify data, ensuring compliance with privacy regulations.

### 2.4. System Security and Access Control

User authentication in Co.Ge. is handled through Laravel’s default session-based system, extended via the Laravel UI package. This setup provides login and registration scaffolding and employs role-based access control to manage permissions. Passwords are securely stored using Bcrypt hashing, and critical routes are protected by middleware to ensure access is restricted to authenticated users. The platform implements CSRF (Cross-Site Request Forgery) protection mechanisms and manages user sessions to safeguard against common web vulnerabilities. In addition, the application includes automated backup routines via the *spatie*/*Laravel-backup* package, which periodically saves the application and database content, contributing to system integrity and data resilience.

### 2.5. Pilot Study

We conducted a pilot study to evaluate the feasibility, technical stability, and data integrity of the Co.Ge. platform. To illustrate the capabilities of the platform, we focus on results from the *Rey Auditory Verbal Learning Test* (AVLT). This task serves as a representative example to demonstrate the collection of both conventional scores and fine-grained behavioral metrics. In particular, we sought to examine the association of gender, education, and clinical status (patient vs. control).

#### 2.5.1. Participants

Participants included adults (age ≥ 18) with and without a clinical condition. Clinical participants were recruited through the Mental Health Department of Ferrara and included individuals with stable psychiatric or neurodegenerative conditions. Non-clinical participants (healthy control participants without current psychiatric conditions or pharmacotherapy) were recruited via online advertisements, flyers, and informal outreach to general hospital and university staff.

Inclusion criteria were (a) age ≥ 18; (b) fluency in Italian, necessary for understanding instructions and accurately completing tests developed in Italian; (c) absence of severe cognitive impairment; (d) clinical stability; and (e) for controls, absence of current psychiatric pharmacotherapy.

Assessments were carried out within the framework of the project “*Cost-effectiveness of innovative, non-pharmacological strategies for early detection, prevention and tailored care of depressive disorders among cancer patients: Transcranial Magnetic Stimulation and Virtual Reality-Based Cognitive Remediation*”, funded by the Italian Ministry of Health under the National Recovery and Resilience Plan (NRRP), #NextGenerationEU, grant number PNRR-MAD-2022-12375899. The study was conducted in accordance with the Declaration of Helsinki and approved by the Ethics Committee of the AUSL of Ferrara.

#### 2.5.2. Procedure

All assessments were conducted in quiet clinical environments by trained professionals using standardized Windows-based laptops (screen size 16″, resolution 1920 × 1080, audio playback enabled) and external microphones to optimize audio capture. All tests were developed and administered in Italian.

Examiners were psychiatrists, psychiatric rehabilitation therapists, and clinical psychologists trained in using the Co.Ge. platform and in standardized cognitive assessment protocols. Examiners followed closely scripted instructions and intervened only to clarify technical aspects of the tasks. Each testing session lasted approximately 20–40 min and included the full Co.Ge. test battery presented in a fixed sequence. Participants interacted directly with the digital platform throughout the session, ensuring uniform delivery and continuous engagement. System stability and user feedback were systematically monitored during piloting to identify potential sources of error (technical, user-related, and contextual) and to guide minor adjustments to improve usability.

#### 2.5.3. Feasibility Outcomes

The pilot phase focused on evaluating interface usability, stability, and user experience. A brief questionnaire was administered to 30 participants following completion of the online testing session. The instrument was developed ad hoc for this exploratory phase. It uses a score of 1 (very bad) to 5 (very good) to evaluate eight core dimensions: efficiency (time, effort, and simplicity in task execution), effectiveness (accuracy and successful task completion), satisfaction (subjective comfort), perceived usefulness and hedonic quality (pleasantness of the interface), perceived load (mental effort to use the software), accessibility (usability for older users), and error robustness (ability to prevent and manage user errors).

#### 2.5.4. Data Collection and Measurements

Demographic, clinical, and screening data were collected using REDCap (14.0.x), a secure, web-based software platform designed to support data acquisition in research contexts [20]. REDCap was used to gather background variables and screening measures for both clinical and non-clinical participants. Screening and diagnostic profiling included structured instruments such as the Brief Psychiatric Rating Scale (BPRS), DSM-5-based clinical interviews, and validated self-report questionnaires assessing depression, anxiety, sleep quality, functioning, trauma, and substance use. Additional neuropsychiatric and functional measures were administered depending on clinical profile and study needs. Cognitive performance and reaction time data were recorded directly through the Co.Ge. platform, which automatically stores structured, task-specific data in a relational MySQL database. While clinical and screening assessments were systematically collected, their results are not the focus of this pilot report and will be analyzed in subsequent studies.

#### 2.5.5. Ethical Approval

Written informed consent was obtained from all participants prior to enrollment. Anonymity was ensured through pseudonymized identifiers and secure data handling procedures. Given the nature of the digital testing environment and the exploratory design, no blinding procedures were implemented.

#### 2.5.6. Statistical Analysis

Descriptive statistics summarized demographic and task data.

First, conventional Linear Mixed Models were employed to analyze immediate recall data from the AVLT, using the number of immediately recalled words as the outcome, testing for the presence of practice effects (effect of five consecutive rounds of word learning) and group differences (clinical vs. controls). Then, we used Bayesian Generalized Linear Mixed Models to explore the distributional properties of reaction times in the AVLT recall data with the brms package [22]. We compared alternative models based on Gaussian, Lognormal and Gamma likelihoods. Each model included clinical group, sex, and a monotonic effect of education level as fixed effects, and a random intercept for participant code. All models were implemented in R using the brms package, with four MCMC chains (4000 iterations each; 1000 warm-up iterations). We evaluated model fit to data with the Leave-One-Out Cross-Validation (LOO) procedure.

All analyses were conducted using R (version 2025.05.0) on Windows systems.

### 2.6. Availability of Materials and Code

The source code for the Co.Ge. platform is available from the corresponding author upon reasonable request for academic use. Due to clinical data protection regulations, the dataset used in this pilot are not publicly available but may be shared in anonymized form upon approval by the ethical board.

## 3. Results

### 3.1. Feasibility

**Figure 1:**
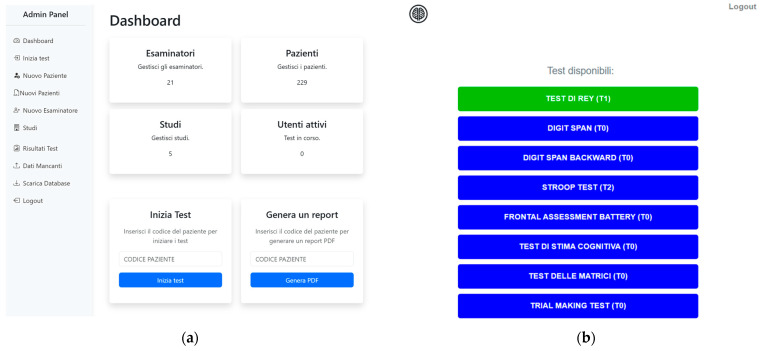
User interface of the Co.Ge. digital platform, Italian version. (**a**) The initial access page, which allows clinicians and researchers to log in and select test sessions, manage users, and configure study parameters. (**b**) Cognitive testing interface for standardized task administration with automated data capture and response monitoring.

Automatic basic data validation (e.g., range checks, timestamp completeness) revealed no systematic issues across sessions. All expected fields were populated, with consistent formatting and logical consistency between entries (e.g., timestamps, round sequences, and response durations). No manual cleaning was required for the variables reported. User language proficiency and pronunciation did not affect the quality of audio recordings, which remained intelligible and correctly aligned with stimulus timing across trials. Timing precision and accuracy were consistently high, with no evidence of system lag or data loss. Examiners reported smooth administration and no major usability issues throughout the testing phase. An overview of the Co.Ge. user interface, including the login dashboard and task administration screen, is provided in Figure 1.

### 3.2. Participants and Completion of Sessions

A total of 123 participants were enrolled in the pilot study, including both clinical and non-clinical adults (age ≥ 18), all of whom completed at least one cognitive assessment using the Co.Ge. platform and were included in the pilot analysis. The sample comprised 81 females (66%) and 42 males (34%), with a mean age of 44.9 years (SD = 17.5). Age was higher among patients (M = 55.3, SD = 13.4) compared to controls (M = 29.2, SD = 9.4). Regarding education, the majority of participants reported either 13 years (36%) or 17 years (41%) of schooling. A smaller proportion reported lower educational levels, such as 3 years (3.3%), 5 years (1.6%), or 8 years (18%), all of which were reported by patients. Based on clinical status, 74 participants (60%) were classified as patients and 49 (40%) as controls (Table 1).

All test sessions were completed without interruptions or technical issues. User experience was generally positive. Effectiveness, accessibility/inclusiveness, and error tolerance—identified a priori as potentially more critical areas—received favorable ratings. The mean score for efficiency was 4.57 (SD = 0.50), followed by satisfaction (4.50, SD = 0.57), effectiveness (3.87, SD = 1.10), perceived usefulness (4.47, SD = 0.57), hedonic quality/stimulation (4.43, SD = 0.57), perceived cognitive load (4.40, SD = 0.62), error tolerance/robustness (3.87, SD = 1.10), and accessibility/inclusiveness (3.77, SD = 1.17). Some participants experienced occasional difficulties, likely related to individual factors such as age, digital familiarity, or cognitive characteristics. Feedback also informed refinements such as supervised administration with headphones and a microphone to reduce distractions and optimize audio quality, and the option to pause or split test sessions to mitigate fatigue effects.

### 3.3. Descriptive Statistics: Rey Auditory Verbal Learning Test

All 123 participants completed the computerized AVLT test. Descriptive inspection of reaction time distributions confirmed a pronounced positive skew, with most response times falling between 10,000 and 30,000 milliseconds and a long tail extending beyond 60,000 ms. A secondary peak was also observed around 35,000 ms.

When stratified by gender, distributions for males and females appeared broadly similar, though female controls showed a more pronounced early peak in faster responses compared to female patients. Among males, the control group displayed a more compact distribution centered around 20,000 ms, whereas patient responses were more variable and extended into longer latencies.

**Figure 2:**
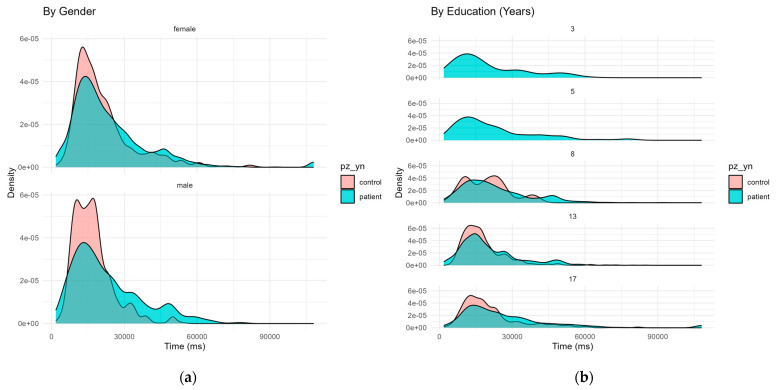
Density plots of reaction times on the Rey Auditory Verbal Learning Test. (**a**) Distributions by gender show largely overlapping patterns, with faster responses among female controls and greater variability among male patients. (**b**) Distributions by education level reveal increasing response consistency and clearer group separation with higher education, while lower education is associated with broader, more skewed patterns.

Stratification by years of education revealed greater dispersion and flattening of the density curves in participants with lower educational attainment (3, 5, and 8 years), especially among patients. In contrast, participants with 13 and 17 years of education showed sharper peaks and more consistent response patterns. Notably, the distributions of patients and controls became more distinct with increasing education level, suggesting a possible moderating effect of education on response consistency and speed (Figure 2). Finally, reaction times correlated weakly but significantly with participant age (r = 0.121, è < 0.001).

### 3.4. Accuracy

Participants’ accuracy in recalling the target words of the Rey auditory–verbal task was analyzed using a generalized linear mixed-effects model with a binomial link. The model included fixed effects for round number, group (control vs. patient), sex, age, and years of education, as well as a random intercept for each participant. A significant main effect of round was observed (β = 0.23, SE = 0.03, *p* < 0.001), indicating improved accuracy over repeated trials across the sample. No significant interaction between round and group was found (β = −0.02, *p* = 0.56), suggesting that learning curves across repetitions did not differ significantly between patients and controls.

The direction of the effect showed a tendency toward lower overall accuracy among patients, but the effect of group did not reach statistical significance (β = −0.46, SE = 0.35, *p* = 0.19). Similarly, no significant effects were found for sex (β = −0.24, *p* = 0.34) or age (β = −0.017, *p* = 0.089), although both showed trends in the expected directions: males and older participants tended to perform slightly worse. Also, years of education exhibited a non-significant positive association with accuracy (β = 0.063, SE = 0.033, *p* = 0.055).

**Figure 3:**
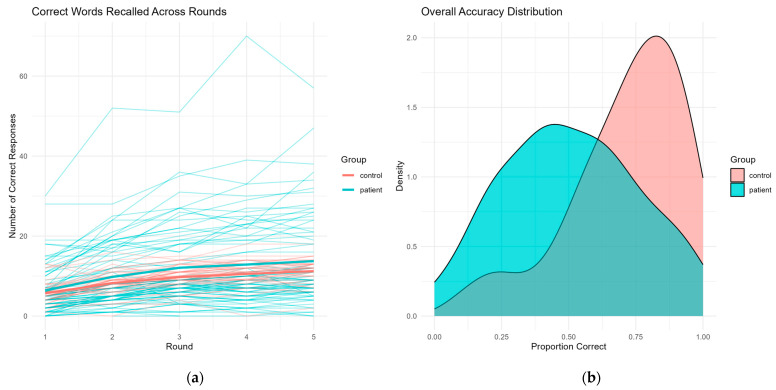
Accuracy patterns in verbal recall performance. (**a**) Individual trajectories of correct responses across rounds reveal higher within-group variability among patients compared to controls. Despite this, both groups show a progressive increase in average performance over trials. (**b**) Density plots of overall accuracy indicate a right-skewed distribution for controls, with most proportions of correct responses clustering around 85–90%, while patients exhibit a broader distribution, with a central tendency below 60%.

These results are visualized in Figure 3. As shown in panel (a), individual trajectories of correct responses across rounds displayed greater variability among patients, while the average accuracy increased steadily in both groups. In panel (b), density plots of overall accuracy distributions highlight a sharper, more right-skewed peak for controls (centered around 85–90% correct responses), in contrast to a broader and flatter distribution among patients, often centered below 60%.

**Figure 4:**
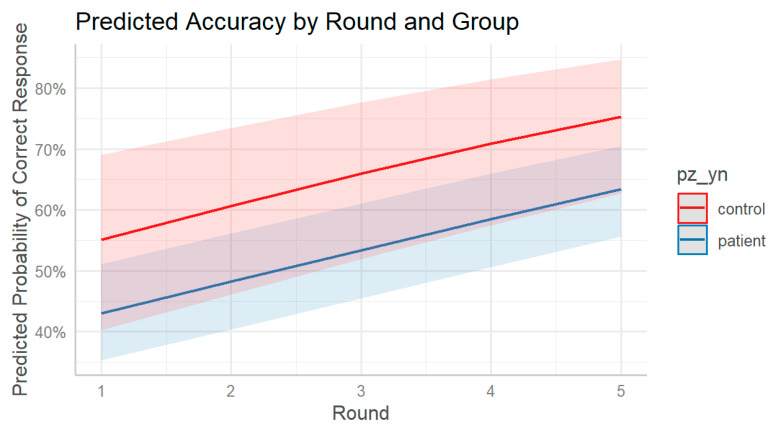
Predicted accuracy by round and group. Marginal predicted probabilities of correct responses across rounds, estimated from the generalized linear mixed-effects model. Both groups show improved accuracy over time, but control participants consistently outperform patients across all rounds. Shaded areas represent 95% confidence intervals.

To further characterize these patterns, predicted marginal probabilities of correct responses were computed for each group across rounds using the fitted model. At round 1, the estimated probability of a correct response was 0.55 for controls and 0.43 for patients. These probabilities increased progressively across rounds, reaching 0.75 for controls and 0.63 for patients by round 5. While both groups showed improvement, the predicted accuracy remained consistently higher in the control group across all repetitions. Notably, the difference between groups was already evident at the beginning of the task and persisted across rounds, despite the absence of a statistically significant interaction effect. These marginal estimates support the presence of a general learning effect and a group-level discrepancy in recall accuracy that remains stable over the task sequence (Figure 4).

### 3.5. Reaction Times

Reaction times were modelled using a linear mixed-effects model including round [1,2,3,4,9], group (control vs. patient), sex, age, and years of education as fixed effects, with a random intercept for participant to account for repeated measures. The model was based on 11,594 observations from 123 individuals and showed adequate fit (REML criterion = 252,710.3). Substantial variability was observed at the subject level, with a standard deviation of 6331 ms, and at the residual level, with a standard deviation of 12,938 ms.

**Figure 5:**
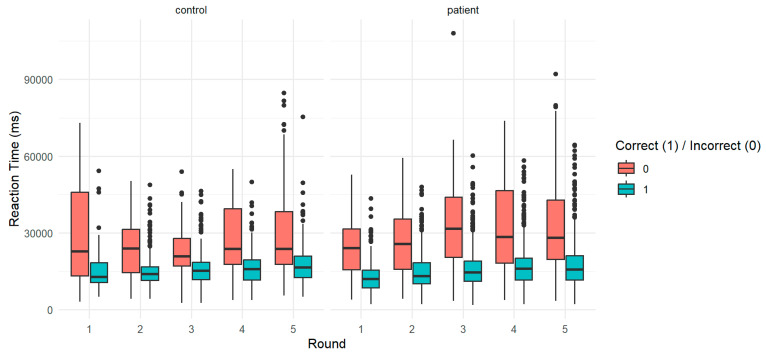
Reaction time by response accuracy and group across rounds. Boxplot of reaction times (in milliseconds) across rounds (1–5), split by response accuracy (correct vs. incorrect) and group (control vs. patient). Correct responses (blue) generally show faster reaction times than incorrect ones (red) in both groups. Patients exhibit greater variability and a progressive increase in reaction times over repeated rounds.

A significant main effect of round (β = 462.7) indicated that reaction times increased over successive repetitions. The main effect of group was also statistically significant (β = −2237.6), with patients exhibiting faster responses than controls at baseline. A significant interaction between round and group (β = 536.5) suggested that the rate of increase in reaction time differed between groups. Age showed a significant positive association with response time (β = 109.0, *p* < 0.05), as did years of education (β = 285.1, *p* < 0.05). The effect of sex (β = −1582.6) was not statistically significant (Figure 5).

**Figure 6:**
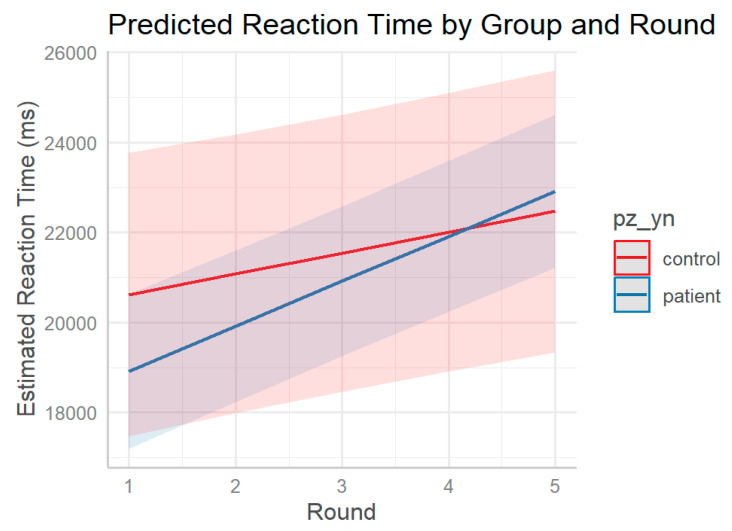
Predicted reaction times across rounds by group. Estimated marginal means of reaction times (in milliseconds) across rounds (1–5) for control and patient groups. While both groups show increasing latency with repetition, patients display a steeper slope, indicating a faster rise in reaction times over rounds. Shaded areas represent 95% confidence intervals.

To explore these trends in more detail, predicted marginal means were computed for each group across rounds At round 1, estimated reaction times were 20,617 ms for controls and 18,916 ms for patients. Across rounds, controls showed a moderate but steady increase in latency, reaching 22,468 ms by round 5, while patient estimates increased more steeply, reaching 22,913 ms by the final round. The crossover observed between groups, with patients initially faster and then slower than controls, reflects distinct temporal patterns across the five repetitions (Figure 6).

### 3.6. Bayesian Hierarchical Modeling of Reaction Times

**Figure 7:**
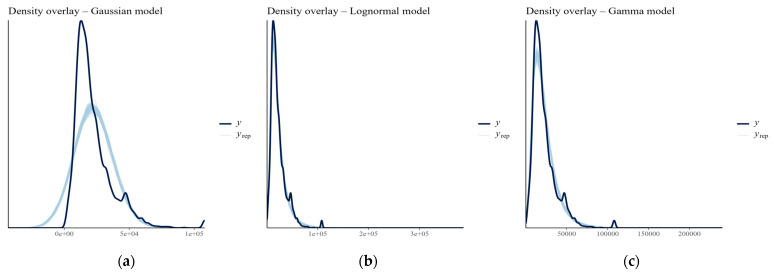
Posterior predictive density overlays for the three candidate models. (**a**) Gaussian model: fails to reproduce the empirical distribution, underestimating both the central peak and the right tail. (**b**) Lognormal model: provides a closer fit to the observed data, capturing the skewness and heavy tail more accurately. (**c**) Gamma model: offers the best alignment with the empirical distribution, closely matching both skewness and dispersion. In all panels, the observed data (dark blue line) are overlaid with 100 posterior predictive distributions (light blue lines) simulated from each model.

All Bayesian GLMM models converged well ($\hat{R}$ ≈ 1.00). The Gaussian model assuming symmetric, homoscedastic residuals showed poorer fit for the right-skewed data and yielded wider credible intervals and a higher residual SD, indicating lower robustness. The lognormal model provided narrower intervals and more stable estimates. The Gamma model slightly outperformed the lognormal model in terms of flexibility and residual dispersion (Table 2). All models yielded good Pareto k values (<0.7) but Leave-One-Out Cross-Validation (LOO-CV) estimates confirmed the Gamma model’s superiority in predictive performance captured individual variation in reaction times (Figure 7).

The model detected a positive effect of round with very high probability (99.9% Probability of Direction, PD), male gender (PD: 93.46%), age (PD: 98.01%), and education (96.3%). Patients had an 83.8% probability of yielding shorter RTs than controls. Also, the interaction between round and patient status was highly significant (PD: 97.9%), so the marginal effect (slope) of round was much larger among patients (mean: 980; 95% CrI: 785; 1174) than among controls (mean: 494; 95% CrI: 225; 759). Thus, the increase in RTs due to practice effects was higher among patients than among controls.

## 4. Discussion

This study introduced Co.Ge., a digital platform for the personalized administration, scoring, and export of cognitive assessment data. In its pilot deployment, Co.Ge. proved feasible and technically stable, as well as user-friendly in both clinical and non-clinical settings. It enabled the automatic collection of structured data, including trial-level reaction times and error patterns, supporting the construction of robust statistical and computational models.

Cognitive testing is a fundamental aspect of psychiatric and medical assessments [3,23,24,25]. Several online platforms for computerized cognitive testing are already available and offer broad capabilities that are similar to those of Co.Ge.—such as millisecond-level reaction time recording and the collection of trial-wise behavioral data. However, these systems typically emphasize experimental precision or clinical standardization, rarely both [26,27,28,29]. In contrast, Co.Ge. was designed from its outset to integrate multiple functions within a single platform: (1) replication of widely used paper-based neuropsychological tasks with minimal ecological loss; (2) structured, high-resolution behavioral logging suitable for advanced statistical and computational analyses; and (3) high compatibility with routine clinical workflows, including supervised administration, longitudinal tracking, and integration with electronic health records. The current findings offer an initial confirmation of the system’s usability, showing that Co.Ge. can capture clinically meaningful variability in cognitive performance while maintaining technical reliability and ease of use in real-world settings.

The pilot study evaluated multiple aspects of the platform’s real-world implementation. Co.Ge. timing mechanisms and verbal response recordings operated as intended, and the system remained stable and user-friendly throughout clinical administration. Automatically recorded response data were consistently captured across tasks, and descriptive performance metrics—including recall scores, error rates, and reaction times—showed interpretable patterns that reflect both normative variability and potential clinical distinctions. These findings offer an initial confirmation of the system’s usability and analytic scope.

Overall, the usability data suggest that Co.Ge. delivers a solid user experience across key domains, with only modest, age-related differences. Participants rated the platform highly for efficiency and satisfaction, and even the constructs we had flagged as higher-risk—effectiveness, accessibility/inclusiveness, and error tolerance—scored well. The few problems we observed occurred almost exclusively in older users: Some found precise mouse control difficult and, on small laptop screens, the default font was hard to read. These issues are typical for senior populations, who often prefer font sizes ≥ 12–14 pt and spacious layouts to reduce visual strain [30]. We have since added an on-screen magnifier and a one-click font-size slider, eliminating complaints in follow-up sessions. A second concern was session length. When all tasks were completed back-to-back, some users reported that the battery of tests took too long. Allowing brief, self-paced breaks or splitting the battery mitigated this aspect. On the positive side, users—especially those with limited digital literacy—praised the voice-guided interface and succinct, plain-language instructions, echoing recent findings that conversational voice assistants enhance perceived ease of use and engagement among older adults [31]. The moderate number of trials and the game-like feel of certain tasks were repeatedly described as “fun”, in line with evidence that hedonic quality boosts adherence in technology-based cognitive assessments [32]. Together with automatic scoring and supervised administration, these adjustments demonstrate that most common sources of error (technical, user-related, or contextual) can be effectively minimized, increasing confidence in the reliability of Co.Ge. Taken together, these results position Co.Ge. as a usable and enjoyable tool that meets diverse accessibility needs while maintaining clinical-grade data quality.

We showcased different levels of usage of Co.Ge. test data with a relatively simple yet clinically important task, the Rey AVLT [33]. Co.Ge. was similarly effective capturing both standard performance metrics and more fine-grained behavioral indicators such as RTs. Here, Bayesian analyses outperformed conventional frequentist GLMMs detecting the effects of education in exploratory analyses [34]. This approach is widely recommended and can be further strengthened by incorporating more advanced generative models (e.g., drift diffusion models, reinforcement learning) [35,36] capable of extracting clinically meaningful latent cognitive parameters with greater potential significance than traditional score-based methods. For example, drift diffusion models can decompose performance in memory or decision-making tasks into latent components such as processing speed, decision thresholds, and attentional fluctuations, thereby providing a richer characterization of cognitive function than raw accuracy or reaction times alone [15,37,38]. It should be noted that Co.Ge. is not currently based on artificial intelligence; its present version relies on standardized task logic, automated scoring, and structured data capture. However, the platform has been designed to support future integration of AI components, including automated speech-to-text transcription and generative computational models, which may further enhance its diagnostic and prognostic value.

The findings highlight the potential of Co.Ge. in advancing high-resolution digital cognitive assessment, in line with recent developments in computational and digital psychiatry, advocating the use of parameter-based individual profiling to support diagnostic and therapeutic decisions [39,40,41,42]. From a clinical perspective, these features could directly support both diagnosis and treatment planning. For example, fine-grained parameters derived from generative models may help distinguish early cognitive impairment from psychiatric conditions with overlapping symptoms (e.g., depression), identify patient-specific cognitive targets for rehabilitation, and monitor treatment response with higher sensitivity than conventional scores. This is especially relevant in light of the principle of equifinality, whereby similar observable outcomes (e.g., memory errors or slowed reaction times) may arise from distinct underlying mechanisms (e.g., neurodegenerative decline versus depression). Generative models are specifically designed to disentangle these mechanisms, allowing clinicians to better interpret what drives apparently similar test results [43,44,45]. The richness of the behavioral data collected by Co.Ge. will enable the investigation of latent cognitive processes such as semantic drifts, attentional fluctuation, executive control, and decision-making dynamics [46,47,48,49,50]. Furthermore, Co.Ge.’s modular architecture supports integration with electronic health records and external databases, making it a flexible tool for both clinical and research contexts. Key features include the ability to design customized cognitive batteries, apply demographic and clinical corrections, and monitor cognitive trajectories over time. This positions Co.Ge. as a promising platform for digital phenotyping and the development of personalized rehabilitation strategies [39,40,41,51,52].

The cognitive domains assessed by Co.Ge.—including memory, attention, executive functions, language, and learning—are transdiagnostic and relevant across different brain disorders, including neurodegenerative (e.g., Alzheimer’s disease, Parkinson’s disease) and neurodevelopmental conditions (e.g., autism spectrum disorder, ADHD). At present, the platform has been tested primarily in clinically stable psychiatric populations, and its use is intended for patients whose condition is compatible with computer-based testing. Broader application to neurodegenerative and neurodevelopmental conditions is plausible but will require further validation studies and potential adaptations, especially for patients at advanced disease stages or during acute decompensation.

This study has limitations: (1) The sample size was relatively limited. Group-level patterns in reaction times and recall trajectories clearly emerged but may need to be confirmed in larger samples, i.e., with cognitive impairments or neurodegenerative conditions. (2) We did not carry out formal equivalence testing for reaction times. Future concurrent validation studies are needed. (3) Longitudinal design is needed to evaluate test–retest reliability and sensitivity to change. Other planned improvements to Co.Ge. include expanding its task library, integrating adaptive testing algorithms, providing multiple language interfaces (the current version is developed only in Italian), and enhancing data processing capabilities.

## 5. Conclusions

We introduced Co.Ge., a digital platform for administering cognitive tasks that seamlessly integrates clinical use with advanced features, including the extraction of detailed behavioral data. This pilot study showed that Co.Ge. is robust and precise yet flexible: This convergence of features—namely, the integration of clinical usability with advanced analytic capabilities—allows it to be useful for routine clinical practice and promote the development of sophisticated generative models for research. Co.Ge. will promote personalized cognitive profiling and digital phenotyping, and pave the way for precision psychiatry and rehabilitation.

## Figures and Tables

**Table 1:** Sociodemographic characteristics of the pilot sample by sex.

Characteristic	Overall, *n* = 123 ^1^	Control, *n* = 49 ^1^	Patient, *n* = 74 ^1^
Sex			
Female	81 (66%)	28 (57%)	53 (72%)
Male	42 (34%)	21 (43%)	21 (28%)
Age	44.9 (17.5)	29.2 (9.4)	55.3 (13.4)
Years of education			
3	4 (3.3%)	0 (0%)	4 (5.4%)
5	2 (1.6%)	0 (0%)	2 (2.7%)
8	22 (18%)	2 (4.1%)	20 (27%)
13	44 (36%)	20 (41%)	24 (32%)
17	51 (41%)	27 (55%)	24 (32%)

^1^ Mean (SD); *n* (%).

**Table 2:** Posterior estimates from the Bayesian Gamma model with log link for reaction time.

Parameter	Median	95% CI	pd	Rhat	ESS
(Intercept)	9.40	[9.05, 9.75]	100%	1.002	1326
round2	0.03	[0.01, 0.04]	99.99%	1.000	11,611
pz_ynpatient	−0.08	[−0.25, 0.08]	83.79%	1.004	1306
sexmale	−0.09	[−0.22, 0.03]	93.46%	1.001	1237
age	0.0051	[0.00, 0.01]	98.01%	1.004	1404
education	0.01	[0.00, 0.03]	96.28%	1.002	1351
round2:pz_ynpatient	0.02	[0.00, 0.03]	97.94%	1.000	11,131

## Data Availability

The source code for the Co.Ge. platform can be obtained from the corresponding author upon reasonable request for academic purposes. Due to clinical data protection policies, the dataset analyzed in this pilot is not publicly accessible but may be made available in anonymized form upon approval by the relevant ethics committee. The copyright application for the Co.Ge. software has been filed and is currently under review.

## Abbreviations

The following abbreviations are used in this manuscript:

API	Application Programming Interface
AVLT	Auditory Verbal Learning Test
BPRS	Brief Psychiatric Rating Scale
BRMS	Bayesian Regression Models using Stan
CEQ	Cannabis Experience Questionnaire
Co.Ge	Cognitive Generative
CSV	Comma-Separated Values
DSM-DP	Department of Mental Health and Addiction Services
DS	Digit Span
DSB	Digit Span Backward
HER	Electronic Health Record
elpd	Expected Log Predictive Density
FDR	False Discovery Rate
Gamma	Gamma regression model (with log link)
GDPR	General Data Protection Regulation
GLMM	Generalized Linear Mixed Model
IDTS	Inventory of Drug-Taking Situations
LOO	Leave-One-Out (Cross-Validation)
Lognormal	Lognormal regression model
LR	Likelihood Ratio
MCMC	Markov Chain Monte Carlo
MVC	Model–View–Controller
MySQL	Structured Query Language (database system)
NRRP	National Recovery and Resilience Plan
ORM	Object-Relational Mapping
PE	Equivalent Score (Punteggio Equivalente)
PRT	Probabilistic Reward Task
R^2^	Coefficient of Determination
REDCap	Research Electronic Data Capture
RT	Reaction Time
SD	Standard Deviation
SQL	Structured Query Language
Stan	Probabilistic Programming Language for Bayesian Inference
TDM	Matrix Test
TMT	Trail Making Test
TSC	Estimation Task
U.O.	Clinical Unit
VR	Virtual Reality

## References

[1] Balsamo M., Imperatori C., Sergi M.R., Belvederi Murri M., Continisio M., Tamburello A., Innamorati M., Saggino A. (2013). Cognitive Vulnerabilities and Depression in Young Adults: An ROC Curves Analysis. Depress. Res. Treat..

[2] Ye S., Sun K., Huynh D., Phi H.Q., Ko B., Huang B., Ghomi R.H. (2022). A Computerized Cognitive Test Battery for Detection of Dementia and Mild Cognitive Impairment: Instrument Validation Study. JMIR Aging.

[3] Murri M.B., Folesani F., Costa S., Morelli A.C., Scillitani V., Guaiana G., Biancosino B., Caruso R., Nanni M.G., Zerbinati L. (2020). Italian Validation of the Screen for Cognitive Impairment in Psychiatry. Community Ment Health J..

[4] Zygouris S., Tsolaki M. (2015). Computerized Cognitive Testing for Older Adults: A Review. Am. J. Alzheimers Dis. Other Demen..

[5] Gualtieri C.T., Johnson L.G. (2006). Reliability and validity of a computerized neurocognitive test battery, CNS Vital Signs. Arch. Clin. Neuropsychol..

[6] Green R.C., Green J., Harrison J.M., Kutner M.H. (1994). Screening for cognitive impairment in older individuals. Validation study of a computer-based test. Arch. Neurol..

[7] Groppell S., Soto-Ruiz K.M., Flores B., Dawkins W., Smith I., Eagleman D.M., Katz Y. (2019). A Rapid, Mobile Neurocognitive Screening Test to Aid in Identifying Cognitive Impairment and Dementia (BrainCheck): Cohort Study. JMIR Aging.

[8] van der Hoek M.D., Nieuwenhuizen A., Keijer J., Ashford J.W. (2019). The MemTrax Test Compared to the Montreal Cognitive Assessment Estimation of Mild Cognitive Impairment. J. Alzheimers Dis..

[9] Chan J.Y.C., Yau S.T.Y., Kwok T.C.Y., Tsoi K.K.F. (2021). Diagnostic performance of digital cognitive tests for the identification of MCI and dementia: A systematic review. Ageing Res. Rev..

[10] Çınar N., Aslan Kendirli S., Florentina Ateş M., Yakupoğlu E., Akbuğa E., Bolu N.E., Karalı F.S., Okluoğlu T., Bülbül N.G., Bayindir E. (2024). Validity and Reliability Study of Online Cognitive Tracking Software (BEYNEX). J. Alzheimer’s Dis. Rep..

[11] Domen A.C., Van De Weijer S.C.F., Jaspers M.W., Denys D., Nieman D.H. (2019). The validation of a new online cognitive assessment tool: The MyCognition Quotient. Int. J. Methods Psychiatr. Res..

[12] Visser L.N., Dubbelman M.A., Verrijp M., Wanders L., Pelt S., Zwan M.D., Thijssen D.H., Wouters H., Sikkes S.A., van Hout H.P. (2021). The Cognitive Online Self-Test Amsterdam (COST-A): Establishing norm scores in a community-dwelling population. Alzheimer’s Dementia Diagn. Assess. Dis. Monit..

[13] Tsoy E., Zygouris S., Possin K.L. (2021). Current State of Self-Administered Brief Computerized Cognitive Assessments for Detection of Cognitive Disorders in Older Adults: A Systematic Review. J. Prev. Alzheimer’s Dis..

[14] Karvelis P., Paulus M.P., Diaconescu A.O. (2023). Individual differences in computational psychiatry: A review of current challenges. Neurosci. Biobehav. Rev..

[15] Haines N., Sullivan-Toole H., Olino T. (2023). From Classical Methods to Generative Models: Tackling the Unreliability of Neuroscientific Measures in Mental Health Research. Biol. Psychiatry Cogn. Neurosci. Neuroimaging.

[16] Chen C.S., Vinogradov S. (2024). Personalized Cognitive Health in Psychiatry: Current State and the Promise of Computational Methods. Schizophr. Bull..

[17] Hessler J., Tucha O., Förstl H., Mösch E., Bickel H. (2014). Age-Correction of Test Scores Reduces the Validity of Mild Cognitive Impairment in Predicting Progression to Dementia. PLoS ONE.

[18] Piccininni M., Rohmann J.L., Wechsung M., Logroscino G., Kurth T. (2023). Should Cognitive Screening Tests Be Corrected for Age and Education? Insights From a Causal Perspective. Am. J. Epidemiol..

[19] O’Connell M.E., Tuokko H., Kadlec H. (2011). Demographic corrections appear to compromise classification accuracy for severely skewed cognitive tests. J. Clin. Exp. Neuropsychol..

[20] Harris P.A., Taylor R., Minor B.L., Elliott V., Fernandez M., O’Neal L., McLeod L., Delacqua G., Delacqua F., Kirby J. (2019). The REDCap consortium: Building an international community of software platform partners. J. Biomed. Inform..

[21] Jound I., Halimi H. (2016). Comparison of Performance Between Raw SQL and Eloquent ORM in Laravel. https://www.diva-portal.org/smash/get/diva2:1014983/FULLTEXT02.

[22] Bürkner P.C. (2017). Advanced Bayesian Multilevel Modeling with the R Package brms. arXiv.

[23] Murri M.B., Folesani F., Costa S., Biancosino B., Colla C., Zerbinati L., Caruso R., Nanni M.G., Purdon S.E., Grassi L. (2020). Screening for cognitive impairment in non-affective psychoses: A comparison between the SCIP and the MoCA. Schizophr. Res..

[24] Folesani F., Murri M.B., Biancosino B., Costa S., Zerbinati L., Caruso R., Nanni M.G., Toffanin T., Ferrara M., Purdon S.E. (2022). The screen for cognitive impairment in psychiatry in patients with borderline personality disorder. Pers. Ment. Health.

[25] Mandini S., Morelli M., Murri M.B., Grassi L., Masotti S., Simani L., Zerbini V., Raisi A., Piva T., Grazzi G. (2022). Adherence to a guided walking program with amelioration of cognitive functions in subjects with schizophrenia even during COVID-19 pandemic. BMC Sports Sci. Med. Rehabil..

[26] Caporusso E., Melillo A., Perrottelli A., Giuliani L., Marzocchi F.F., Pezzella P., Giordano G.M. (2025). Current limitations in technology-based cognitive assessment for severe mental illnesses: A focus on feasibility, reliability, and ecological validity. Front. Behav. Neurosci..

[27] Feenstra H.E.M., Vermeulen I.E., Murre J.M.J., Schagen S.B. (2017). Online cognition: Factors facilitating reliable online neuropsychological test results. Clin. Neuropsychol..

[28] Holmlund T.B., Foltz P.W., Cohen A.S., Johansen H.D., Sigurdsen R., Fugelli P., Bergsager D., Cheng J., Bernstein J., Rosenfeld E. (2019). Moving psychological assessment out of the controlled laboratory setting: Practical challenges. Psychol. Assess..

[29] Swapnajeet S., Sandeep G. (2022). Technology-based Neurocognitive Assessment of the Elderly: A Mini Review. Consort Psychiatr..

[30] Hou G., Anicetus U., He J. (2022). How to design font size for older adults: A systematic literature review with a mobile device. Front. Psychol..

[31] Shade M., Yan C., Jones V.K., Boron J. (2025). Evaluating Older Adults’ Engagement and Usability With AI-Driven Interventions: Randomized Pilot Study. JMIR Form. Res..

[32] Wu M., Feng J., Sun R., Zhang S., Zhang Y., Yang F., Zhang X., Ye Y., Gong N., Liao J. (2025). Validity and usability for digital cognitive assessment tools to screen for mild cognitive impairment: A randomized crossover trial. J. Neuroeng. Rehabil..

[33] Possemis N., ter Huurne D., Banning L., Gruters A., Van Asbroeck S., König A., Linz N., Tröger J., Langel K., Blokland A. (2024). The Reliability and Clinical Validation of Automatically-Derived Verbal Memory Features of the Verbal Learning Test in Early Diagnostics of Cognitive Impairment. J. Alzheimer’s Dis..

[34] McElreath R. (2018). Statistical Rethinking: A Bayesian Course with Examples in R and Stan.

[35] Ossola P., Antonucci C., Meehan K.B., Cain N.M., Ferrari M., Soliani A., Marchesi C., Clarkin J.F., Sambataro F., De Panfilis C. (2021). Effortful control is associated with executive attention: A computational study. J. Pers..

[36] Montemitro C., Ossola P., Ross T.J., Huys Q.J.M., Fedota J.R., Salmeron B.J., di Giannantonio M., Stein E.A. (2024). Longitudinal changes in reinforcement learning during smoking cessation: A computational analysis using a probabilistic reward task. Sci. Rep..

[37] Torous J., Staples P., Barnett I., Sandoval L.R., Keshavan M., Onnela J.P. (2018). Characterizing the clinical relevance of digital phenotyping data quality with applications to a cohort with schizophrenia. npj Digit. Med..

[38] Frässle S., Yao Y., Schöbi D., Aponte E.A., Heinzle J., Stephan K.E. (2018). Generative models for clinical applications in computational psychiatry. WIREs Cogn. Sci..

[39] Paulus M.P., Huys Q.J.M., Maia T.V. (2016). A Roadmap for the Development of Applied Computational Psychiatry. Biol. Psychiatry Cogn. Neurosci. Neuroimaging.

[40] Durstewitz D., Huys Q.J.M., Koppe G. (2021). Psychiatric Illnesses as Disorders of Network Dynamics. Biol. Psychiatry Cogn. Neurosci. Neuroimaging.

[41] Weichart E.R., Darby K.P., Fenton A.W., Jacques B.G., Kirkpatrick R.P., Turner B.M., Sederberg P.B. (2021). Quantifying mechanisms of cognition with an experiment and modeling ecosystem. Behav. Res..

[42] Torous J., Bucci S., Bell I.H., Kessing L.V., Faurholt-Jepsen M., Whelan P., Carvalho A.F., Keshavan M., Linardon J., Firth J. (2021). The growing field of digital psychiatry: Current evidence and the future of apps, social media, chatbots, and virtual reality. World Psychiatry.

[43] Poile C., Safayeni F. (2016). Using Computational Modeling for Building Theory: A Double Edged Sword. JASSS.

[44] Cicchetti D., Rogosch F.A. (1996). Equifinality and multifinality in developmental psychopathology. Dev. Psychopathol..

[45] Carozza S., Akarca D., Astle D. (2023). The adaptive stochasticity hypothesis: Modeling equifinality, multifinality, and adaptation to adversity. Proc. Natl. Acad. Sci. USA.

[46] Huys Q.J.M., Browning M., Paulus M.P., Frank M.J. (2021). Advances in the computational understanding of mental illness. Neuropsychopharmacology.

[47] Rutledge R.B., Chekroud A.M., Huys Q.J. (2019). Machine learning and big data in psychiatry: Toward clinical applications. Curr. Opin. Neurobiol..

[48] Schurr R., Reznik D., Hillman H., Bhui R., Gershman S.J. (2024). Dynamic computational phenotyping of human cognition. Nat. Hum. Behav..

[49] Khaleghi A., Mohammadi M.R., Shahi K., Nasrabadi A.M. (2022). Computational Neuroscience Approach to Psychiatry: A Review on Theory-driven Approaches. Clin. Psychopharmacol. Neurosci..

[50] Zemla J.C., Cao K., Mueller K.D., Austerweil J.L. (2020). SNAFU: The Semantic Network and Fluency Utility. Behav. Res..

[51] Torous J., Staples P., Onnela J.P. (2015). Realizing the Potential of Mobile Mental Health: New Methods for New Data in Psychiatry. Curr. Psychiatry Rep..

[52] Wiecki T.V., Poland J., Frank M.J. (2015). Model-based cognitive neuroscience approaches to computational psychiatry: Clustering and classification. Clin. Psychol. Sci..

